# Association of Exposure to Fine-Particulate Air Pollution and Acidic Gases with Incidence of Nephrotic Syndrome

**DOI:** 10.3390/ijerph15122860

**Published:** 2018-12-14

**Authors:** Shih-Yi Lin, Wu-Huei Hsu, Cheng-Li Lin, Cheng-Chieh Lin, Chih-Hsueh Lin, I-Kuan Wang, Chung-Y. Hsu, Chia-Hung Kao

**Affiliations:** 1 Graduate Institute of Biomedical Sciences and School of Medicine, College of Medicine, China Medical University, 404 Taichung, Taiwan; oasisbestonly@yahoo.com.tw (S.-Y.L.); Hsuwh@mail.cmuh.org.tw (W.-H.H.); cclin@mail.cmuh.org.tw (C.-C.L.); hsuc@mail.cmuh.org.tw (C.-Y.H.); 2 Division of Nephrology and Kidney Institute, China Medical University Hospital, 404 Taichung, Taiwan; ikwang@mail.cmuh.org.tw; 3 Department of Chest Medicine, China Medical University Hospital, 404 Taichung, Taiwan; 4 Management Office for Health Data, China Medical University Hospital, 404 Taichung, Taiwan; orangechengli@gmail.com; 5 College of Medicine, China Medical University, 404 Taichung, Taiwan; 6 Department of Family Medicine, China Medical University Hospital, 404 Taichung, Taiwan; 7 Department of Geriatrics, China Medical University Hospital, 404 Taichung, Taiwan; d5496@mail.cmuh.org.tw; 8 Department of Nuclear Medicine and PET Center, China Medical University Hospital, 404 Taichung, Taiwan; 9 Department of Bioinformatics and Medical Engineering, Asia University, 413 Taichung, Taiwan

**Keywords:** air pollution, PM_2.5_, nephrotic syndrome, retrospective study

## Abstract

*Background*: Air pollution has been associated with autoimmune diseases. Nephrotic syndrome is a clinical manifestation of immune-mediated glomerulopathy. However, the association between nephrotic syndrome and air pollution constituents remains unknown. We conducted this nationwide retrospective study to investigate the association between PM_2.5_ and nephrotic syndrome. *Methods*: We used the Longitudinal Health Insurance Database (LHID) and the Taiwan Air Quality-Monitoring Database (TAQMD). We combined and stratified the LHID and the TAQMD data by residential areas of insurants linked to nearby air quality-monitoring stations. Air pollutant concentrations were grouped into four levels based on quartile. Univariable and multivariable Cox proportional hazard regression models were applied. *Findings*: Relative to Q1-level SO_2_, subjects exposed to the Q4 level were associated with a 2.00-fold higher risk of nephrotic syndrome (adjusted HR = 2.00, 95% CI = 1.66–2.41). In NOx, relative to Q1 NOx concentrations, the adjusted HRs of nephrotic syndrome risk were 1.53 (95% CI = 1.23–1.91), 1.30 (95% CI = 1.03–1.65), and 2.08 (95% CI = 1.69–2.56) for Q2, Q3, and Q4 levels, respectively. The results revealed an increasing trend for nephrotic syndrome risk correlating with increasing levels of NO, NO_2_, and PM_2.5_ concentrations. *Interpretation*: High concentrations of PM_2.5_, NO, NO_2_, and SO_2_ are associated with increased risk of nephrotic syndrome.

## 1. Introduction

Nephrotic syndrome—massive proteinuria as a result of heterogeneous dysfunction of the glomerulus—has detrimental effects on long-term renal function [1]. Although nephrotic syndrome has been divided into mechanistic categories, the majority of nephrotic syndrome remains idiopathic and multifaceted [2]. The estimated annual incidence of nephrotic syndrome in healthy children is two to seven new cases per 100,000 population [3,4]. Both racial and environmental factors have been hypothesized to be involved in the increased susceptibility to nephrotic syndrome [5]. Studies have shown that black people had more prevalence of focal segmental glomerular sclerosis and Asian people had more chances of minimal change disease, compared with Caucasian population [6,7]. For age classifications, the pathology of nephrotic syndrome differed between childhood and adulthood; meanwhile, nephrotic syndrome in adults had much more diverse group of diseases, less chance of minimal change disease, poor response to steroid treatment, and longer time to remission [8]. Furthermore, neonatal and childhood nephrotic syndrome had been found to be more frequently linked with monogenic defects [9]. Studies have identified potential environmental triggers of nephrotic syndrome, including exposure to mercury and its salts [10], secondary syphilis [11], aminonucleosides [12], and organic chemicals [13]. Genetic and environmental factors might affect phenotypic variability among nephrotic syndrome cases [14]. Thus, properly identifying potential environmental triggers of nephrotic syndrome is a key strategy in preventing the development of nephrotic syndrome.

Most identified environmental pathways of nephrotic syndrome have been through ingestion. However, recent studies have also shown that inhalation may be a potential pathway for nephrotic syndrome [15]. For example, Xu et al. showed that air pollution in Mainland China was associated with risk of membrane nephropathy [15]. Xu et al. found that the adjusted odds for membranous nephropathy increased 13% annually over the 11-year study period, whereas the proportions of other major glomerulopathies remained stable [15]. Furthermore, they reported that each 10 μg/m^3^ increase in PM_2.5_ concentration associated with 14% higher odds for MN in regions with PM_2.5_ concentration >70 μg/m^3^ [15]. Although researchers have comparatively analyzed average PM_2.5_ concentrations and the occurrence of membrane nephropathy, data on the association between the composition of PM_2.5_ and nephrotic syndrome are lacking. In recent decades, Taiwan has been under a transition period entailing the discovery and implementation of alternative energy resources for nuclear energy [16]. Currently, the majority of resources of electricity in Taiwan are from the Taichung coal-burning power plant, which is the top ten largest power plants in the world [17]. Furthermore, Taiwan has worse air pollution with increasing concentrations of PM_2.5_ than before [17]. However, the association between air pollution PM_2.5_ and nephrotic syndrome had not been investigated. Previous study has shown that SO_2_ and NOx acid gases were potential inflammation triggers and associated with autoimmune disease [18]. Since triggering autoimmunity is one of the pathogenic pathways of nephrotic syndrome [19], we used the National Health Insurance Research Database (NHIRD) to conduct a retrospective cohort study investigating whether PM_2.5_ and acidic gases are associated with the occurrence of nephrotic syndrome in Taiwan.

## 2. Materials and Methods

### 2.1. Data Source

We conducted a population-based cohort study using the Longitudinal Health Insurance Database (LHID) and the Taiwan Air Quality-Monitoring Database (TAQMD). The details of the LHID and TAQMD have been well documented in previous studies [20,21]. We combined and stratified the LHID and the TAQMD by residential areas of insurants linked to nearby air quality-monitoring stations. Diagnoses associated with hospital use were coded according to the International Classification of Diseases, Ninth Revision, Clinical Modification (ICD-9-CM). The participants were assigned to residential districts based on the clinic from where they most frequently sought treatment for acute upper respiratory infection. Thus, residential area was determined based on the clinic and hospital of the insurant when treated for acute upper respiratory tract infections (ICD-9-CM code 460). This study was approved by the Research Ethics Committee of China Medical University and Hospital in Taiwan (CMUH104-REC2-115-CR2).

### 2.2. Sampled Participants

This study cohort was selected based on where the insurants lived on 1 January 2000, which was designated as the study’s index date. We excluded insurants with a history of nephrotic syndrome (ICD-9-CM code 581) before the index date. The endpoint for follow-up was the date of withdrawal from the program, development of nephrotic syndrome (ICD-9-CM code 581), or 31 December 2011. A daily average air pollutant concentration was calculated from 1998 until the end of the observation year for each study subject. Air pollutant concentrations were grouped into four levels based on quartile: SO_2_ concentration (first quartile [Q1]: <3.38, second quartile [Q2]: 3.38–4.31, third quartile [Q3]: 4.32–6.03, and fourth quartile [Q4]: >6.03 ppb), NOx concentration (Q1: <23.4, Q2: 23.4–31.9, Q3: 32.0–38.6, and Q4: >38.6 ppb), NO concentration (Q1: <5.16, Q2: 5.16–8.57, Q3: 8.58–11.5, and Q4: >11.5 ppb), NO_2_ concentration (Q1: <18.2, Q2: 18.2–23.6, Q3: 23.7–27.5, and Q4: >27.5 ppb), and PM_2.5_ concentration (Q1: <29.5, Q2: 29.5–33.2, Q3: 33.3–41.2, and Q4: >41.2 μg/m^3^). The confounding factors considered in this study were gender, age, monthly income, and urbanization level. The Institutes stratified Taiwan into 7 urbanization levels, based on not only scores of population density (people/km^2^) but also proportion of higher education, elderly and agricultural population, and the number of physicians per 100,000 people in each area. In our study, Level 1 represents areas with a higher population density and socioeconomic status, and Level 7 the lowest. Because few people lived in more rural areas of Levels 4–7, we therefore grouped these 4 types of areas into a Level “4”.

### 2.3. Statistical Analysis

Category variables, such as sex, monthly income, urbanization level, and outcome, are presented as numbers and percentages, and differences were assessed using a chi-squared test. The incidence density rate of nephrotic syndrome (per 10,000 person-years) was calculated at different air pollutant concentration levels. Univariable and multivariable Cox proportional hazard regression models were used to estimate the hazard ratios (HRs) and 95% confidence intervals (CIs) for nephrotic syndrome in Levels Q2–Q4 for air pollutant concentration relative to the lowest level (Q1). The multivariable model was adjusted for age, sex, monthly income, and urbanization level. To address the concern of constant proportionality, we examined the proportional hazard model assumption using a test of scaled Schoenfeld residuals. There was no significant relationship between Schoenfeld residuals for SO_2_, NOx, NO, and NO_2_ and follow-up time (*p*-value = 0.75, 0.48, 0.48, 0.66, respectively) in the model evaluating the nephrotic syndrome. In the model evaluating the nephrotic syndrome risk throughout overall follow-up period, results of the test revealed a significant relationship between Schoenfeld residuals for PM_2.5_ and follow-up time (*p*-value < 0.001), suggesting the proportionality assumption was violated. In the subsequent analyses, we stratified the follow-up duration to deal with the violation of proportional hazard assumption. Variables found to be significant in the univariable analysis were further included in the multivariable analysis. We further tested the interaction between air pollutant and confounders by including a cross-product term in the model. If the interaction was significant, we also put in the model for adjustment. All analyses were conducted using SAS software Version 9.4 (SAS Institute Inc., Cary, NC, USA), and the significance level was set at a two-tailed P less than 0.05.

## 3. Results

The present study included the follow-up data on 161,970 residents. After the 12-year follow-up, 776 participants developed nephrotic syndrome (Table 1). The mean age at enrollment was 40.5 ± 14.6, and men accounted for 43.8% of all participants. Most participants lived in moderately urbanized areas (32.5%). The daily average SO_2_, NOx, NO, NO_2_, and PM_2.5_ concentrations were 4.98 ± 2.41 ppb, 36.3 ± 35.1 ppb, 11.0 ± 10.2 ppb, 22.6 ± 6.57 ppb, and 34.8 ± 8.76 μg/m^3^. 

Participants resided in the most highly urbanized towns exposed to the Q4 level including SO_2_, NOx, NO, NO_2_, and PM_2.5_ (Table 2).

Table 3 shows the risk of nephrotic syndrome among levels of air pollutant concentrations. Relative to Q1-level SO_2_, the subjects exposed at the Q4 level were associated with a 2.00-fold higher risk of nephrotic syndrome (adjusted HR = 2.00, 95% CI = 1.66–2.41). For NOx, relative to Q1 NOx concentrations, the adjusted HRs of nephrotic syndrome risk were 1.32 (95% CI = 1.00–1.73) for Q2 levels. For NO, relative to Q1 NO concentrations, the adjusted HRs of nephrotic syndrome risk were 1.34 (95% CI = 1.02–1.77) for Q2 levels. The data revealed an increasing trend for nephrotic syndrome risk correlating with increasing levels of PM_2.5_ concentrations. The adjusted HRs of nephrotic syndrome risk were 1.33 (95% CI = 0.91–1.93), 1.96 (95% CI = 1.08–3.55), and 2.53 (95% CI = 1.08–5.94) for subjects exposed to Q1-level PM_2.5_, Q2-level PM_2.5_, and Q3-level PM_2.5_, respectively.

Table 4 shows the risk of nephrotic syndrome among levels of PM_2.5_ concentrations stratified by follow-up period. Relative to Q1-level PM_2.5_, the subjects exposed at the Q4 level were associated with a 4.23-fold higher risk of nephrotic syndrome (adjusted HR = 4.23, 95% CI = 1.02–1.76) for the follow-up period ≤6 years. For PM_2.5_, relative to Q1 PM_2.5_ concentrations, the adjusted HRs of nephrotic syndrome risk were 1.65 (95% CI = 1.04–2.61), 2.16 (95% CI = 1.02–4.55) for Q2 level and Q3 level. 

## 4. Discussion

This study clearly demonstrated that increased exposure to increasing quartile concentrations of PM_2.5_, SO_2_, NOx, and NO were associated with increased risk of nephrotic syndrome in Taiwan. However, the underlying mechanism remains to be investigated; nevertheless, several pathways might explain this finding. First, whatever the pathologic pattern of nephrotic syndrome is, the mechanism of nephrotic syndrome is believed to be associated with autoimmunity [22]. Autoimmunity triggers the production of circulating autoantibodies. Such autoantibodies may target either the components of the glomerulus directly, forming the immune complex in situ, or ubiquitous antigens of systemic autoimmune disease, forming circulating complexes that are deposited in the glomerulus [23]. A previous study found that changes to the charge of the immune complex altered the glomerulus barrier and may contribute to nephrotic syndrome [24]. Air pollution has been closely related to chronic airway diseases, including asthma and chronic obstructive pulmonary disease [25]. Air pollution has recently also been associated with systemic autoimmune diseases [26], intestinal diseases [27], and diabetes mellitus [28], in which alterations in autoimmunity play a role. Air pollution has been found to induce oxidative-stress DNA damage [29], and such DNA damage could contribute to the development of autoimmunity [30]. Van Eeden et al. found that air pollution increased levels of interleukin (IL)-6, IL-1β, macrophage inflammatory protein-1α (MIP-1α), granulocyte macrophage colony-stimulating factor (GM-CSF), and circulating levels of IL-1β, IL-6, and GM-CSF [31]. These cytokines have previously been associated with the development of nephrotic syndrome [32,33,34]. Thus, air pollution PM_2.5_ might increase systemic oxidative-stress [18] and inflammatory response, triggering autoimmunity and thus altering the barrier of the glomerulus to increase occurrences of nephrotic syndrome.

Furthermore, increasing quartile concentrations of SO_2_, NOx, NO_2_, and NO were associated with higher incidences of nephrotic syndrome. Bernatsky et al. found that industrial emitters of sulfur dioxide were associated with increased anticitrullinated antibody (ACPA) positivity [35]. In addition, Franze et al. noted that abundant nitrogen oxide air pollutants promoted protein nitration [36]. Nitrated protein, which is an inflammatory-associated product [37], is associated with loss of immune tolerance [38] and triggers autoimmunity [39,40]. We hypothesize that the aforementioned pathways may also contribute to the increased incidences of nephrotic syndrome. This is the first study to examine the association between nephrotic syndrome and increasing quartile concentrations of SO_2_, NOx, NO_2_, and NO.

Several similarities and differences should be mentioned between our study and the study of Xu et al. [15]. First, this study is a 12-year nationwide study, which is slightly longer that of Xu et al. [15]. Second, we used quartile concentrations to examine correlations with the incidence of nephrotic syndrome. This strategy could alleviate possible bias, such as biopsy rates and urbanization. Furthermore, our results are robust irrespective of whether the trends of nephrotic syndrome are increasing or decreasing. Third, because the development of nephrotic syndrome would need an incubation period, correlating the quartile concentrations of air pollution with nephrotic syndrome would be more appropriate to examine the association between air pollution and nephropathy. Fourth, we analyzed the trends for quartile air pollution concentrations with nephrotic syndrome. Our results demonstrate that a clear dose–response relationship exists between air pollution and nephrotic syndrome. Finally, Taiwan researchers have found an association of osteoporosis and rheumatic diseases with air pollution PM_2.5_ [18,19]. Our and the above studies showed that air pollution has threatened the general health of Taiwan citizens [18,19]. Since Taiwan is a country with the top three highest incidence of renal disease in the world [41], our study might provide implications for public health impacts and policy, such as shortening the period of finding alternative power sources and setting national alarm systems for air quality. 

Several limitations of this study should also be mentioned. First, the information regarding family history, past atypical infection, viral infection, specific diet preference, over-the-counter medication use (including nonsteroidal anti-inflammatory drugs), and exposure to gold salts, which are potential risk factors of nephrotic syndrome, are unavailable in the NHIRD. Second, we analyzed the serial concentration of fine-particulate air pollution (PM_2.5_), but information about individual exposure to fine-particulate PM_2.5_ was unavailable. Some participants may have worn an N95 mask, used an air cleaner while inside, or avoided outdoor activities during periods of serious air pollution, thus lessening personal contact with PM_2.5_. Additionally, some participants might have had increased PM_2.5_ exposure due to their occupations and working environments. Because there would be variations in pollutant levels among these cities, bias from regional differences would exist and possible baseline bias may also exist in this study. Third, information about single-nucleotide polymorphisms, including variants of the nephrin gene (NPHS1) [42], GPC5 [43], PLCE1 [44], and PLA2R1 [45] are unavailable in NHIRD.

## 5. Conclusions

In conclusion, this study reported an association between fine-particulate air pollution PM_2.5_ and occurrences of nephrotic syndrome, suggesting that air pollution might cause nephrotic syndrome. Additional studies are required to investigate whether avoiding exposure to PM_2.5_ could lessen the risks or delay the progression of nephrotic syndrome.

## Figures and Tables

**Table 1:** Baseline demographics and exposure of air pollutants in Taiwan.

N = 161,970		n	%
Gender	Men	70,948	43.8
	Women	91,022	56.2
Age, years	mean, SD	40.5	14.6
Urbanization level	1 (highest)	55,898	34.5
	2	52,644	32.5
	3	27,407	16.9
	4 (lowest)	26,020	16.1
Exposure of air pollutants			
SO_2_ level (daily average, ppb)	mean, SD	4.98	2.41
	Min	0.44	
	Lower quartile	3.38	
	Median	4.32	
	Upper quartile	6.03	
	90th percentile	8.68	
	Maximum	14.1	
NOx level (daily average, ppb)	mean, SD	36.3	35.1
	Min	1.65	
	Lower quartile	23.4	
	Median	32.0	
	Upper quartile	38.6	
	90th percentile	54.8	
	Maximum	426.7	
NO level (daily average, ppb)	mean, SD	11.0	10.2
	Min	0.32	
	Lower quartile	5.16	
	Median	8.58	
	Upper quartile	11.5	
	90th percentile	24.1	
	Maximum	60.4	
NO_2_ level (daily average, ppb)	mean, SD	22.6	6.57
	Min	0.85	
	Lower quartile	18.2	
	Median	23.7	
	Upper quartile	24.5	
	90th percentile	30.6	
	Maximum	38.6	
PM_2.5_ level (daily average, μg/m^3^)	mean, SD	34.8	8.76
	Min	1.00	
	Lower quartile	29.5	
	Median	33.3	
	Upper quartile	41.2	
	90th percentile	47.7	
	Maximum	113.2	
Outcome			
Nephrotic syndrome	Yes	776	0.48
Follow-up time, years	mean, SD	11.7	0.99

The urbanization level was categorized by the population density of the residential area into 4 levels, with level 1 as the most urbanized and level 4 as the least urbanized. SO_2_, sulfur dioxide; NOx, nitrogen oxides; NO, nitrogen monoxide; NO_2_, nitrogen dioxide; PM, particulate matter; PM_2.5_, particles with aerodynamic diameter < 2.5 μm; SD, standard deviation.

**Table 2:** Baseline urbanization level among quartiles of daily average concentration of air pollutants in Taiwan.

Air Pollutant Concentration	Quartile 1 (Q1) (lowest)		Quartile 2 (Q2)		Quartile 3 (Q3)		Quartile 4 (Q4) (highest)		* *p*-Value
N = 161,970	n	(%)	n	(%)	n	(%)	n	(%)	
**Sulfur dioxide (SO_2_)**									<0.001
Urbanization level									
1 (highest)	12,662	(29.9)	13,928	(36.4)	19,315	(41.6)	9993	(28.6)	
2	13,428	(31.7)	11,114	(29.1)	13,820	(29.8)	14,282	(40.8)	
3	4829	(11.4)	5479	(14.3)	8887	(19.2)	8212	(23.5)	
4 (lowest)	11,411	(27.0)	7721	(20.2)	4389	(9.46)	2499	(7.14)	
**Nitrogen oxides (NOx)**									<0.001
Urbanization level									
1 (highest)	8667	(22.4)	10,836	(25.2)	10,950	(32.5)	25,445	(54.6)	
2	13,095	(33.9)	15,717	(36.5)	12,480	(37.0)	11,352	(24.4)	
3	4777	(12.4)	8540	(19.9)	7358	(21.8)	6732	(14.5)	
4 (lowest)	12,101	(31.3)	7932	(18.4)	2934	(8.70)	3053	(6.55)	
**Nitrogen monoxide (NO**)									<0.001
Urbanization level									
1 (highest)	8329	(21.0)	9967	(24.3)	16,250	(45.0)	21,352	(47.4)	
2	13,887	(35.0)	13,634	(33.2)	11,368	(31.5)	13,755	(30.5)	
3	4255	(10.7)	10,186	(24.8)	5471	(15.2)	7495	(16.6)	
4 (lowest)	13,244	(33.4)	7261	(17.7)	3035	(8.40)	2480	(5.50)	
**Nitrogen dioxide (NO_2_)**									<0.001
Urbanization level									
1 (highest)	8313	(23.1)	11,056	(23.4)	21,609	(45.0)	14,920	(48.7)	
2	10,953	(30.4)	18,620	(39.4)	14,202	(29.6)	8869	(29.0)	
3	5454	(15.1)	7740	(16.4)	9075	(18.9)	5138	(16.8)	
4 (lowest)	11,308	(31.4)	9850	(20.8)	3152	(6.56)	1710	(5.58)	
**Particulate matter (PM_2.5_)**									<0.001
Urbanization level									
1 (highest)	22,062	(47.4)	14,301	(40.2)	10,754	(27.5)	8781	(21.6)	
2	13,032	(28.0)	10,350	(29.1)	13,593	(34.7)	15,669	(38.5)	
3	5443	(11.7)	6153	(17.3)	5749	(14.7)	10,062	(24.7)	
4 (lowest)	6054	(13.0)	4733	(13.3)	9063	(23.1)	6170	(15.2)	

* Chi-square test. The urbanization level was categorized by the population density of the residential area into 4 levels, with level 1 as the most urbanized and level 4 as the least urbanized. The daily average air pollutant concentrations were categorized into 4 groups based on quartiles for each air pollutant.

**Table 3:** Differences in nephrotic syndrome incidences and associated HRs in participants exposed to various daily average concentrations of air pollutants.

Pollutant Levels	N	Event	PY	IR	cHR	95%CI	aHR	95%CI
SO_2_^1^								
Q1	42,331	184	493,552	3.73	Ref.		Ref.	
Q2	38,242	129	448,116	2.88	0.77	(0.62, 0.97)	0.80	(0.64, 1.00)
Q3	46,411	179	543,975	3.29	0.88	(0.72, 1.08)	0.97	(0.79, 1.20)
Q4	34,986	284	407,059	6.98	1.87	(1.56,2.25) ***	2.00	(1.66, 2.41) ***
NOx ^2^								
Q1	38,640	126	451,937	2.79	Ref.		Ref.	
Q2	43,026	207	503,906	4.11	1.47	(1.18, 1.84) ***	1.32	(1.00, 1.73) *
Q3	33,722	154	395,166	3.90	1.40	(1.10, 1.77) **	1.15	(0.79, 1.68)
Q4	46,582	289	541,693	5.34	1.91	(1.55, 2.36) ***	1.41	(0.89, 2.22)
NO ^3^								
Q1	39,715	127	465,107	2.73	Ref.		Ref.	
Q2	41,048	201	480,251	4.19	1.66	(1.33, 2.07) ***	1.34	(1.02, 1.77) *
Q3	36,125	151	423,750	3.56	1.64	(1.28, 2.09) ***	1.12	(0.76, 1.64)
Q4	45,082	297	523,594	5.67	2.49	(2.01, 3.09) ***	1.47	(0.92, 2.35)
NO_2_^4^								
Q1	36,028	127	421,548	3.01	Ref.		Ref.	
Q2	47,267	228	553,336	4.12	1.37	(1.10, 1.70) **	1.23	(0.94, 1.61)
Q3	48,038	208	563,416	3.69	1.23	(0.98, 1.53)	1.02	(0.69, 1.51)
Q4	30,637	213	354,402	6.01	2.00	(1.61, 2.49) ***	1.35	(0.78, 2.33)
PM_2.5_^5^								
Q1	46,591	129	545,526	2.36	Ref.		Ref.	
Q2	35,537	126	416,539	3.02	1.28	(1.00, 1.64) *	1.33	(0.91, 1.93)
Q3	39,160	212	457,761	4.63	1.96	(1.57, 2.44) ***	1.96	(1.08, 3.55) *
Q4	40,682	309	472,877	6.53	2.77	(2.26, 3.40) ***	2.53	(1.08, 5.94) *

Q1 = first quartile. Q2 = second quartile. Q3 = third quartile. Q4 = fourth quartile. PY = person-years. IR = incidence rate, (per 10,000 person-years). cHR = crude hazard ratio. aHR = adjusted hazard ratio. CI = confidence interval. Ref. = reference group. ^1^ Adjusted HR was calculated by Cox proportional hazard regression and adjusted for age, sex, monthly income, and urbanization level. ^2^ Adjusted HR was calculated by Cox proportional hazard regression and adjusted for age, sex, monthly income, and urbanization level, and interaction of NOx and monthly income. ^3^ Adjusted HR was calculated by Cox proportional hazard regression and adjusted for age, sex, monthly income, urbanization level, and interaction of NO and urbanization level. ^4^ Adjusted HR was calculated by Cox proportional hazard regression and adjusted for age, sex, monthly income, urbanization level, and interaction of NO and monthly income. ^5^ Adjusted HR was calculated by Cox proportional hazard regression and adjusted for age, sex, monthly income, urbanization level, interaction of PM_2.5_ and age, interaction of PM_2.5_ and sex, interaction of PM_2.5_ and monthly income, and interaction of PM_2.5_ and urbanization level. * *p* < 0.05; ** *p* < 0.01; *** *p* < 0.001.

**Table 4:** Differences in nephrotic syndrome incidences and associated HRs in participants exposed to daily average concentrations of PM_2.5_ stratify by follow-up period.

	cHR	95%CI	aHR	95%CI
PM_2.5_				
Follow-up period ≤ 6				
Q1	Ref.		Ref.	
Q2	0.63	(0.38, 1.03)	0.73	(0.36, 1.44)
Q3	1.19	(0.80, 1.78)	1.53	(0.55, 4.22)
Q4	3.31	(2.38, 4.59) ***	4.23	(1.02, 17.6) *
Follow-up period > 6				
Q1	Ref.		Ref.	
Q2	1.67	(1.25, 2.23) ***	1.65	(1.04, 2.61) *
Q3	2.42	(1.85, 3.15) ***	2.16	(1.02, 4.55) *
Q4	2.45	(1.88, 3.19) ***	1.93	(0.66, 5.68)

Q1 = first quartile. Q2 = second quartile. Q3 = third quartile. Q4 = fourth quartile. cHR = crude hazard ratio. aHR = adjusted hazard ratio of a multivariate analysis, after adjustment for age, sex, monthly income, urbanization level, interaction of PM_2.5_ and age, interaction of PM_2.5_ and sex, interaction of PM_2.5_ and monthly income, and interaction of PM_2.5_ and urbanization level. CI = confidence interval. Ref. = reference group. * *p* < 0.05; *** *p* < 0.001.

## References

[1] Atkins R.C., Briganti E.M., Lewis J.B., Hunsicker L.G., Braden G., Champion de Crespigny P.J., DeFerrari G., Drury P., Locatelli F., Wiegmann T.B. (2005). Proteinuria reduction and progression to renal failure in patients with type 2 diabetes mellitus and overt nephropathy. Am. J. Kidney Dis..

[2] Bierzynska A., McCarthy H.J., Soderquest K., Sen E.S., Colby E., Ding W.Y., Nabhan M.M., Kerecuk L., Hegde S., Hughes D. (2017). Genomic and clinical profiling of a national nephrotic syndrome cohort advocates a precision medicine approach to disease management. Kidney Int..

[3] Tapia C., Bashir K. (2018). Nephrotic Syndrome.

[4] Hull R.P., Goldsmith D.J.A. (2008). Nephrotic syndrome in adults. BMJ.

[5] Feehally J., Kendell N.P., Swift P.G., Walls J. (1985). High incidence of minimal change nephrotic syndrome in Asians. Arch. Dis. Child..

[6] Korbet S.M., Genchi R.M., Borok R.Z., Schwartz M.M. (1996). The racial prevalence of glomerular lesions in nephrotic adults. Am. J. Kidney Dis..

[7] Banh T.H., Hussain-Shamsy N., Patel V., Vasilevska-Ristovska J., Borges K., Sibbald C., Lipszyc D., Brooke J., Geary D., Langlois V. (2016). Ethnic Differences in Incidence and Outcomes of Childhood Nephrotic Syndrome. Clin. J. Am. Soc. Nephrol..

[8] Canetta P.A.A., Radhakrishnan J. (2015). The Evidence-Based Approach to Adult-Onset Idiopathic Nephrotic Syndrome. Front. Pediatr..

[9] Benoit G., Machuca E., Antignac C. (2010). Hereditary nephrotic syndrome: A systematic approach for genetic testing and a review of associated podocyte gene mutations. Pediatr. Nephrol..

[10] Kazantzis G., Schiller K.F., Asscher A.W., Drew R.G. (1962). Albuminuria and the nephrotic syndrome following exposure to mercury and its compounds. Q. J. Med..

[11] Braunstein G.D., Lewis E.J., Galvanek E.G., Hamilton A., Bell W.R. (1970). The nephrotic syndrome associated with secondary syphilis. An immune deposit disease. Am. J. Med..

[12] Frenk S., Antonowicz I., Craig J.M., Metcoff J. (1955). Experimental nephrotic syndrome induced in rats by aminonucleoside; renal lesions and body electrolyte composition. Proc. Soc. Exp. Biol. Med..

[13] Bell G.M., Gordon A.C., Lee P., Doig A., MacDonald M.K., Thomson D., Anderton J.L., Robson J.S. (1985). Proliferative glomerulonephritis and exposure to organic solvents. Nephron.

[14] Bullich G., Trujillano D., Santín S., Ossowski S., Mendizábal S., Fraga G., Madrid Á., Ariceta G., Ballarín J., Torra R. (2015). Targeted next-generation sequencing in steroid-resistant nephrotic syndrome: mutations in multiple glomerular genes may influence disease severity. Eur. J. Hum. Genet..

[15] Xu X., Wang G., Chen N., Lu T., Nie S., Xu G., Zhang P., Luo Y., Wang Y., Wang X. (2016). Long-Term Exposure to Air Pollution and Increased Risk of Membranous Nephropathy in China. J. Am. Soc. Nephrol..

[16] Shih Y.-H., Shi N.-X., Tseng C.-H., Pan S.-Y., Chiang P.-C. (2016). Socioeconomic costs of replacing nuclear power with fossil and renewable energy in Taiwan. Energy.

[17] Chen T.-L. (2018). Air Pollution Caused by Coal-fired Power Plant in Middle Taiwan. Int. J. Energy Power Eng..

[18] Gawda A., Majka G., Nowak B., Marcinkiewicz J. (2017). Air pollution, oxidative stress, and exacerbation of autoimmune diseases. Cent.-Eur. J. Immunol..

[19] Shao X.S., Yang X.Q., Zhao X.D., Li Q., Xie Y.Y., Wang X.G., Wang M., Zhang W. (2009). The prevalence of Th17 cells and FOXP3 regulate T cells (Treg) in children with primary nephrotic syndrome. Pediatr. Nephrol..

[20] Chang K.H., Chang M.Y., Muo C.H., Wu T.N., Hwang B.F., Chen C.Y., Lin T.H., Kao C.H. (2015). Exposure to air pollution increases the risk of osteoporosis: A nationwide longitudinal study. Medicine (Baltimore).

[21] Chang K.H., Hsu C.C., Muo C.H., Hsu C.Y., Liu H.C., Kao C.H., Chen C.Y., Chang M.Y., Hsu Y.C. (2016). Air pollution exposure increases the risk of rheumatoid arthritis: A longitudinal and nationwide study. Environ. Int..

[22] Lewis M.G., Loughridge L.W., Phillips T.M. (1971). Immunological studies in nephrotic syndrome associated with extrarenal malignant disease. Lancet.

[23] Mastroianni-Kirsztajn G., Hornig N., Schlumberger W. (2015). Autoantibodies in renal diseases—Clinical significance and recent developments in serological detection. Front. Immunol..

[24] Harvey S.J., Jarad G., Cunningham J., Rops A.L., van der Vlag J., Berden J.H., Moeller M.J., Holzman L.B., Burgess R.W., Miner J.H. (2007). Disruption of glomerular basement membrane charge through podocyte-specific mutation of agrin does not alter glomerular permselectivity. Am. J. Pathol..

[25] Peng R.D., Chang H.H., Bell M.L., McDermott A., Zeger S.L., Samet J.M., Dominici F. (2008). Coarse particulate matter air pollution and hospital admissions for cardiovascular and respiratory diseases among Medicare patients. JAMA.

[26] Farhat S.C., Silva C.A., Orione M.A., Campos L.M., Sallum A.M., Braga A.L. (2011). Air pollution in autoimmune rheumatic diseases: A review. Autoimmun. Rev..

[27] Beamish L.A., Osornio-Vargas A.R., Wine E. (2011). Air pollution: An environmental factor contributing to intestinal disease. J. Crohn’s Colitis.

[28] Eze I.C., Hemkens L.G., Bucher H.C., Hoffmann B., Schindler C., Künzli N., Schikowski T., Probst-Hensch N.M. (2015). Association between ambient air pollution and diabetes mellitus in Europe and North America: systematic review and meta-analysis. Environ. Health Perspect..

[29] Risom L., Møller P., Loft S. (2005). Oxidative stress-induced DNA damage by particulate air pollution. Mutat. Res..

[30] Li Y., Zhao M., Yin H., Gao F., Wu X., Luo Y., Zhao S., Zhang X., Su Y., Hu N. (2010). Overexpression of the growth arrest and DNA damage-induced 45alpha gene contributes to autoimmunity by promoting DNA demethylation in lupus T cells. Arthritis Rheumatol..

[31] van Eeden S.F., Tan W.C., Suwa T., Mukae H., Terashima T., Fujii T., Qui D., Vincent R., Hogg J.C. (2001). Cytokines involved in the systemic inflammatory response induced by exposure to particulate matter air pollutants (PM(10)). Am. J. Respir. Crit. Care Med..

[32] Horii Y., Muraguchi A., Iwano M., Matsuda T., Hirayama T., Yamada H., Fujii Y., Dohi K., Ishikawa H., Ohmoto Y. (1989). Involvement of IL-6 in mesangial proliferative glomerulonephritis. J. Immunol..

[33] Kim S.D., Park J.M., Kim I.S., Choi K.D., Lee B.C., Lee S.H., Hong S.J., Jin S.Y., Lee H.J., Hong M.S. (2004). Association of IL-1beta, IL-1ra, and TNF-alpha gene polymorphisms in childhood nephrotic syndrome. Pediatr. Nephrol..

[34] Lloyd C.M., Minto A.W., Dorf M.E., Proudfoot A., Wells T.N., Salant D.J., Gutierrez-Ramos J.C. (1997). RANTES and monocyte chemoattractant protein-1 (MCP-1) play an important role in the inflammatory phase of crescentic nephritis, but only MCP-1 is involved in crescent formation and interstitial fibrosis. J. Exp. Med..

[35] Bernatsky S., Smargiassi A., Joseph L., Awadalla P., Colmegna I., Hudson M., Fritzler M.J. (2017). Industrial air emissions, and proximity to major industrial emitters, are associated with anti-citrullinated protein antibodies. Environ. Res..

[36] Franze T., Weller M.G., Niessner R., Pöschl U. (2005). Protein nitration by polluted air. Environ. Sci. Technol..

[37] Ohmori H., Kanayama N. (2005). Immunogenicity of an inflammation-associated product, tyrosine nitrated self-proteins. Autoimmun. Rev..

[38] Gauba V., Grünewald J., Gorney V., Deaton L.M., Kang M., Bursulaya B., Ou W., Lerner R.A., Schmedt C., Geierstanger B.H. (2011). Loss of CD4 T-cell-dependent tolerance to proteins with modified amino acids. Proc. Natl. Acad. Sci. USA.

[39] Ahsan H. (2013). 3-Nitrotyrosine: A biomarker of nitrogen free radical species modified proteins in systemic autoimmunogenic conditions. Hum. Immunol..

[40] Khan F., Ali R. (2006). Antibodies against nitric oxide damaged poly L-tyrosine and 3-nitrotyrosine levels in systemic lupus erythematosus. J. Biochem. Mol. Biol..

[41] Tsai S.-Y., Tseng H.-F., Tan H.-F., Chien Y.-S., Chang C.-C. (2009). End-stage renal disease in Taiwan: a case-control study. J. Epidemiol..

[42] Lahdenkari A.T., Kestilä M., Holmberg C., Koskimies O., Jalanko H. (2004). Nephrin gene (NPHS1) in patients with minimal change nephrotic syndrome (MCNS). Kidney Int..

[43] Okamoto K., Tokunaga K., Doi K., Fujita T., Suzuki H., Katoh T., Watanabe T., Nishida N., Mabuchi A., Takahashi A. (2011). Common variation in GPC5 is associated with acquired nephrotic syndrome. Nat. Genet..

[44] Hinkes B., Wiggins R.C., Gbadegesin R., Vlangos C.N., Seelow D., Nürnberg G., Garg P., Verma R., Chaib H., Hoskins B.E. (2006). Positional cloning uncovers mutations in PLCE1 responsible for a nephrotic syndrome variant that may be reversible. Nat. Genet..

[45] Lv J., Hou W., Zhou X., Liu G., Zhou F., Zhao N., Hou P., Zhao M., Zhang H. (2013). Interaction between PLA2R1 and HLA-DQA1 variants associates with anti-PLA2R antibodies and membranous nephropathy. J. Am. Soc. Nephrol..

